# Development of a Mitochondrial Permeability Transition‐Driven Necrosis‐Related Prognostic Signature in Cervical Cancer: Integrating Bulk Transcriptomic and Single‐Cell Data

**DOI:** 10.1002/cam4.71094

**Published:** 2025-08-01

**Authors:** Jiaojiao Niu, Sreenivasan Sasidharan

**Affiliations:** ^1^ School of Biological Engineering Xinxiang University Xinxiang City Henan Province China; ^2^ Institute for Research in Molecular Medicine (INFORMM) Universiti Sains Malaysia Gelugor Pulau Pinang Malaysia

**Keywords:** cell communication, cervical carcinoma, nomogram, prognostic genes, regulator network

## Abstract

**Background:**

Cervical cancer (CC) is a prevalent gynecological malignancy with notable heterogeneity. The role of mitochondrial permeability transition (MPT)‐driven necrosis, a form of cell death due to mitochondrial dysfunction, in CC progression and prognosis is poorly understood and represents a promising therapeutic target for cancers. This study aimed to create a new prognostic signature linked to MPT‐driven necrosis, improving CC prediction and prognosis.

**Methods:**

This study utilized the GSE63514, TCGA‐CESC, CGCI‐HTMCP‐CC, and GSE197641 transcriptome datasets. Firstly, the GSE63514 dataset was utilized to identify differentially expressed genes (DEGs). Differentially expressed MPT‐driven necrosis‐related genes (DE‐MRGs) were obtained by intersecting DEGs with MRGs. Regression analyses were performed to identify genes significantly associated with prognosis. A prognostic model was established in TCGA‐CESC, followed by independent validation and nomogram construction. Additional analyses included immune infiltration, enrichment analysis, and drug susceptibility based on high‐ and low‐risk groups. Finally, cell communication analysis was performed to investigate interactions between key cell types.

**Results:**

A total of 156 DE‐MRGs were identified. Regression analyses identified three prognostic genes (*ICOS*, *MMP3*, and *POSTN*) to construct a prognostic risk signature. Then, risk score was an independent prognostic factor for CC, and a nomogram demonstrated effective predictive accuracy for CC survival outcomes. The risk signature was linked to immune‐associated processes such “Antigen processing and presentation” and immune cell infiltration, especially M0 macrophages and CD8 T cells. Cell communication analysis uncovered a strong interaction between endothelial cells and monocytes. To validate the molecular mechanisms, qRT‐PCR, cell proliferation, and wound healing assays were performed. Functional tests showed that MMP3 and POSTN knockdown drastically reduced CC cell growth and migration.

**Conclusion:**

This study developed a novel prognostic risk signature based on *ICOS*, *MMP3*, and *POSTN*. MMP3 and POSTN knockdown significantly decrease CC cell growth and migration, highlighting their potential as therapeutic targets.

**Abbreviations**
CC: Cervical cancer
ceRNA: competing endogenous RNA
CESC: cervical squamous cell carcinoma and endocervical adenocarcinoma
CypD: critically dependent on cyclophilin D
DCA: decision curve analysis
DE‐MRGs: differentially expressed MRGs
EMT: epithelial‐mesenchymal transition
ESTIMATE: Estimation of Stromal and Immune cells in Malignant Tumors using the Expression
GEO: Gene Expression Omnibus
GO: Gene Ontology
GSEA: Gene Set Enrichment Analysis
HR‐HPV: high‐risk human papillomavirus
IC50: inhibitory concentration
ICOSLG: ICOS ligand
ICP: immune check point
ICPs: immune checkpoints
IMM: inner mitochondrial membrane
KEGG: Kyoto Encyclopedia of Genes and Genomes
MMP: mitochondrial membrane potential
MPT: mitochondrial permeability transition
MPTP: mitochondrial permeability transition pore
MRGs: mitochondrial‐related genes
OS: overall survival
PCA: principal component analysis
RCD: regulated cell death
RT‐qPCR: reverse transcription‐quantitative polymerase chain reaction
scRNA‐seq: single‐cell RNA sequencing
TCGA‐CESC: the cancer genome atlas cervical squamous cell carcinoma and endocervical adenocarcinoma
TCGA‐GDC: TCGA‐genomic data commons
TFs: transcription factors
TIDE: tumor immune dysfunction and exclusion

## 1 Introduction

Cervical cancer (CC) is a leading gynecological malignancy, with approximately 530,000 new cases and 270,000 deaths annually worldwide, highlighting its significant impact on global health [1, 2]. Most CC cases are classified as squamous cell carcinoma and adenocarcinoma (CESC) [2]. Persistent infection with oncogenic high‐risk human papillomavirus (HR‐HPV) is a critical factor in the pathogenesis of CC [3]. Despite advancements in immunization and early detection through cytological screening, CC remains a significant healthcare challenge, particularly in low‐to‐middle‐income countries where access to these preventative measures is limited [4]. Traditional treatment options, including surgery, chemotherapy, and radiotherapy, along with emerging targeted therapies and immunotherapies, are highly effective for early‐stage CC. However, treatment options are limited and less effective for patients with metastatic or recurrent disease, resulting in poorer outcomes [5, 6]. The molecular mechanisms underlying CC, particularly those contributing to treatment resistance, remain poorly understood, leading to a grim long‐term prognosis for many patients [3, 7]. Therefore, identifying accurate and reliable prognostic risk models is crucial for improving prognostic predictions, personalizing treatment strategies, and ultimately enhancing CC patient outcomes.

Recent breakthroughs in understanding novel cell death mechanisms—such as ferroptosis, necroptosis, and pyroptosis—have opened up promising new therapeutic strategies for cancer treatment by exploiting distinct pathways that trigger tumor cell death. Within this context, mitochondrial permeability transition (MPT)‐driven necrosis, a specialized type of non‐apoptotic regulated cell death (RCD), has emerged as a particularly noteworthy focus of research, largely because of its ability to circumvent conventional apoptosis resistance commonly observed in cancer cells. This form of necrosis is characterized by cellular lysis triggered by intracellular disturbances, including oxidative stress and excessive cytosolic Ca^2+^ accumulation, with cyclophilin D (CypD) playing a pivotal and indispensable role in the process [8]. MPT was first described in the 1970s when researchers discovered Ca^2+^‐mediated membrane transition in mitochondria, a key event that disrupts mitochondrial function and leads to cell death [9, 10]. MPT is characterized by the permeabilization of the inner mitochondrial membrane (IMM), permitting the passage of large solutes, which results in a rapid loss of mitochondrial membrane potential (MMP), ATP depletion, and the osmotic breakdown of mitochondrial membranes [8, 11]. This phenomenon is mediated by a supramolecular structure known as the mitochondrial permeability transition pore (MPTP), which forms at the junctions between the inner and outer mitochondrial membranes and with cyclophilin D (CypD), which is the only confirmed essential component [8, 12, 13]. MPT‐driven necrosis has been implicated in various pathological processes, including cardiac and degenerative diseases [14]. Recent studies have also highlighted its critical role in tumorigenesis, impacting cancers such as melanoma [15], glioma [16], colorectal cancer [17], prostate cancer [18], hepatocellular carcinoma [19] and laryngeal squamous cell carcinoma [20]. Targeting MPT‐driven necrosis presents a promising therapeutic approach for refractory cancers. For example, Zhang, Cheng, Shi, Song, Chen, Chen, Yao, Zhang, and Cai [21] demonstrated that a sphingosine kinase inhibitor SKI‐V induces the programmed necrosis cascade in CC cells by promoting the interaction between mitochondrial p53, cyclophilin‐D, and ANT1. Despite these findings, the prognostic significance of MPT‐driven necrosis in CC remains unclear.

Recent advancements in bioinformatics have revolutionized the integration of single‐cell RNA sequencing (scRNA‐seq) with transcriptomic analysis, greatly enhancing our ability to investigate biological mechanisms at single‐cell resolution. The progress in scRNA‐seq technology has significantly improved our capacity to characterize tumor heterogeneity and intercellular networks at this resolution [22]. This technology shows considerable promise for the identification of novel prognostic biomarkers and therapeutic targets [23]. Moreover, integrating single‐cell and transcriptome data improves the accuracy of prognostic models, thereby facilitating more precise risk stratification and personalized treatment strategies for cancer patients [23]. For example, the Fudan University team applied scRNA‐seq to classify cervical cancer into four molecular subtypes: hypoxic (S‐H), proliferative (S‐P), differentiated (S‐D), and immune‐active (S‐I). Among these, the immune‐active subtype demonstrated the best prognosis, whereas the hypoxic subtype had the poorest prognosis [24].

In this study, we developed an innovative prognostic signature based on three key prognostic genes by integrating bulk transcriptomic and single‐cell data. We validated the robustness, precision, and independent prognostic value of the model. Additionally, we explored functional enrichment, immune cell infiltration, immunotherapy efficacy, and drug sensitivity across different risk groups. We also constructed a TF‐miRNA‐mRNA network and conducted a comprehensive cell communication analysis. Ultimately, we validated the mRNA levels of the three prognostic genes using qRT‐PCR in four cell lines. Our findings highlight the predictive potential of MRGs in CC, providing new insights into therapeutic targets.

## 2 Materials and Methods

### 2.1 Data Origination

This study integrated multi‐omics datasets and functional gene sets from public repositories: bulk transcriptomic data included the discovery cohort GSE63514 (28 cervical carcinoma [CC] vs. 24 normal tissues; Gene Expression Omnibus, https://www.ncbi.nlm.nih.gov/geo/), the training set TCGA‐CESC (*n* = 304; TCGA‐Genomic Data Commons, https://portal.gdc.cancer.gov/), and the validation set CGCI‐HTMCP‐CC (*n* = 117; CGCI, https://portal.gdc.cancer.gov/). Single‐cell transcriptomics utilized GSE197641 (1 CC vs. 1 precancerous sample). Gene sets comprised mitochondrial permeability transition (MPT)‐related necrosis markers (M17902/M3873/M16257, 39 genes; GSEA), apoptosis/necrosis/pyroptosis‐associated genes (87/72/28 genes; GSEA), and ferroptosis‐related genes (*n* = 259; FerrDb, http://www.datjar.com:40013/bt2104/).

### 2.2 Identification and Investigation of Differentially Expressed Genes (DEGs) and Their Functions

The “limm” package (v3.54.0) [25] was used to perform differential expression analysis between the CC and normal groups in the GSE63514 dataset. DEGs were identified based on an adjusted *p* < 0.05 and |log2FoldChange| > 1. Single‐sample GSEA (ssGSEA) was employed to calculate the scores for each gene in the MRG sets. Data was then partitioned into high and low‐scoring groups according to the median scores. Survival differences between these groups in TCGA‐CESC were analyzed. The same “limm” package parameters were used to identify DEGs between the high and low‐scoring groups. DEGs common to both analyses were considered differentially expressed MRGs (DE‐MRGs). Gene Ontology (GO) and Kyoto Encyclopedia of Genes and Genomes (KEGG) were performed using the “clusterProfile” (v4.2.2) [26] package with *p* < 0.05.

### 2.3 Evaluation and Verification of the Risk Model

Univariate Cox regression analysis based on DE‐MRGs was performed using the “surviva” package (version 0.4.9) [27] with HR ≠ 1, *p* < 0.05 [28]. The Proportional hazards (PH) assumption test and stepwise regression were used to further filter prognostic genes. Risk scores were calculated for TCGA‐CESC patients using the formula:

$$\text{risk score}=\sum_{n=1}^{n}\left(\text{coefi}*\mathrm{Xi}\left(i=1-n\right)\right)$$

Based on the median risk score, the optimal threshold for classifying patients into high‐ and low‐risk groups was determined. Kaplan–Meier (KM) survival analysis was performed using the “survmine” package (version 0.4.9) [27] to compare survival between these two groups. Gene expression heat maps were plotted using the “pheatma” package (version1.0.12) [29] and receiver operating characteristic (ROC) curves were plotted using the “survivalRO” package (version 1.0.3.1) [30]. Similar steps were followed for the validation set. A gene–gene interaction (GGI) network was constructed by GeneMANIA (https://genemania.org/) to identify the functional enrichment of prognostic genes and their interacting genes [31].

### 2.4 Independent Prognosis and Analysis of Clinical Characteristics

In TCGA‐CESC, risk score and other pathological indicators were used to establish a prognostic model. Five variables, risk score, age, and T/N/M stages, were subjected to univariate Cox regression analysis. After the PH assumption test and multivariate Cox regression analysis, significant factors were incorporated into a nomogram constructed using the “rm” package (version 6.5–0) [27]. Calibration curves and decision curve analysis (DCA) were plotted. Spearman correlation analysis was performed to understand the interactions between prognostic genes and other cell death forms. GSEA was conducted using the “clusterProfile” package (version 4.2.2) with the reference gene set “c2.kegg.v7.4.symbols” [32].

### 2.5 Analysis of the Linkage Analysis of Risk Scores and Immunotherapy

The correlation between differential immune checkpoint (ICP) genes was examined. KM analysis was used to explore survival differences of ICP genes between the two risk groups. Tumor immune dysfunction and exclusion (TIDE) analysis was employed to evaluate the potential response to immune checkpoint blockade therapy. Differences in TIDE scores and their association with risk scores were investigated.

### 2.6 Construction of Immune Cell Characterization

To assess the immune microenvironment of CC in TCGA‐CESC, the Estimation of Stromal and Immune cells in Malignant Tumors using the Expression data (ESTIMATE) algorithm was employed to compute the stromal, immune, and ESTIMATE scores. The Wilcoxon rank sum test was applied to identify differences in the scores between risk groups. The CIBERSORT algorithm analyzed variations in immune cell populations between groups. The “corrplo” package (version 0.92) was used to map the connection matrix of 22 tumor‐infiltrating immune cells [33]. Pearson correlation (|correlation coefficient (cor)| > 0.3, *p* < 0.05) was performed to explore the link between differential immune cells with risk scores. Cox regression analysis identified immune cells associated with overall survival (OS) and KM analysis [34] used activated mast cell scores as an independent risk assessment index to explore the survival changes of immune cells.

### 2.7 Analysis of Drug Sensitivity and the Building of Regulatory Networks

The predictive power of multiple targeted therapeutics was assessed using the “pRRopheti” package (v0.5) [35], which estimates the half‐maximal inhibitory concentration (IC_50_) values based on gene expression profiles. Differences in IC_50_ between risk groups were analyzed using the “oncoPredi” package (version 0.1) [36]. Subcellular localization of prognostic genes was determined using mRNALocater (http://bio‐bigdata.cn/mRNALocater/result/). Functional correlations between prognostic genes were detected using the “GOSemSi” package (version 2.24.0) [37], and Pearson's test explored associations between prognostic genes. MicroRNAs (miRNAs) related to prognostic genes were predicted using the “multiMi” package (version 1.20.0) [38] and the MiRDB database (https://mirdb.org/). The Starbase database predicted lncRNAs based on miRNA. A competing endogenous RNA (ceRNA) network was plotted using Cytoscape software (version 3.9.1). Transcription factors (TFs) for miRNA were predicted using the TransmiR database (http://www.cuilab.cn/transmir), and the TF‐miRNA‐mRNA network was constructed with Cytoscape software (version 3.9.1).

### 2.8 Cellular Annotation and Cellular Communication

Integrating bulk transcriptomic and single‐cell data to formulate a novel signature based on three prognostic genes represents a significant advancement. This approach allows for a more comprehensive understanding of the molecular mechanisms underlying disease progression and provides a unique perspective on potential therapeutic targets. GSE197641 was processed via the “Seura” package (version 4.1.0) [39], and cells with fewer than 200 genes and cells with fewer than 3 covered genes were removed. Quality control criteria included nFeature_RNA greater than 300 and less than 5000, nCount_RNA less than 25,000, and percent.mt less than 10%. Data normalization was performed using LogNormalize, and highly variable genes were filtered using the “FindVariableFeature” function. Principal component analysis (PCA) was used to detect consistency and outliers. Linear dimensionality reduction identified the best dimensionality values for cell clustering, and unsupervised cluster analysis was implemented via “FindNeighbor” and “FindCluster” functions. The “FindAllMarker” function recognized important marker genes for different clusters, and cell types were named accordingly. The “Single” package (version 2.0.0) [40] assisted in cluster annotation and illustrating the expression levels of prognostic genes in different cell types. Cell communication analysis based on key cells was performed using the “CellCha” package (version 1.6.1) [41].

### 2.9 Single‐Cell Analysis of T Cell Subsets

Use the subset function to filter out T cells type cells and create a new Seurat object for these cells. Apply the global scaling normalization method LogNormalize to standardize each sample, then use the vst method of the FindVariableFeatures function to identify genes that exhibit a high coefficient of variation among cells, thereby selecting the top 2000 genes with significant fluctuations. Execute PCA analysis on single‐cell samples using the RunPCA function, calculate the P‐value of each gene in each principal component using the JackStraw function, and use ScoreJackStraw to evaluate the significance strength of the principal components. We display the standard deviation of each principal component after dimensionality reduction and sort the information represented by each principal component (percentage of variance). Next, perform dimensionality reduction analysis on the samples using the umap algorithm (resolution set to 0.3) based on the first four principal components. Using the “FindAllMarkers” function in the R package “Seurat” (only.pos = TRUE, min.pct = 0.25, logfc.threshold = 0.25) on samples from the GSE197641 dataset, identify cell marker genes. Integrating the marker genes obtained from the “FindAllMarkers” function, the provided marker genes, and information from the reference [24, 42]. Using the DotPlot function in the R package “Seurat,” we display the expression levels of prognostic genes (ICOS, MMP3, POSTN) across different T‐cell subtypes. The CellChat software package was used to analyze cell communication of annotated cell clusters for the study of cell interactions and signaling mechanisms. Utilize the “netVisual_heatmap” function in the “CellChat” package to plot network diagrams and heatmaps to display the results. Finally, to understand the developmental trajectory of key cells and the expression changes of prognostic genes within these cells, conduct pseudotime trajectory analysis on the key cells using the monocle package, and visualize the results with the “plot_cell_trajectory” function.

### 2.10 Reverse Transcription‐Quantitative Polymerase Chain Reaction (RT‐qPCR)

RT‐qPCR was conducted to quantify the expression of prognostic genes in cervical cancer (CC) cell lines and to assess the knockdown efficiency of MMP3 and POSTN. Total RNA was extracted using TRIzol reagent (Thermo Fisher Scientific, China) according to the manufacturer's instructions. For prognostic gene profiling, RNA was isolated from ECt1/E6E7, HeLa, SiHa, and CaSki cells cultured to 70%–90% confluence, with three biological replicates per group. For gene silencing analysis, RNA was extracted from HeLa cells 48 h after transfection with siRNAs targeting MMP3 or POSTN. cDNA was synthesized from 1 μg of total RNA using the SureScript First‐Strand cDNA Synthesis Kit (Servicebio, China), following the manufacturer's protocol. Quantitative PCR was conducted using 2× SYBR Green Master Mix (Bio‐Rad, USA). Primers were synthesized by ThermoFisher (Table S1), and GAPDH was used as the internal reference. The $2^{-\Delta \Delta c_{t}}$ method was used to calculate relative gene expression levels.

### 2.11 RNA Interference

Small interfering RNA (siRNA) targeting MMP3 and POSTN mRNA (ON‐TARGETplus SMARTpool siRNA, Thermo Fisher Scientific, Rockford, IL, USA) was transfected into cells using DharmaFECT 1 transfection reagent (Thermo Fisher Scientific) according to the manufacturer's instructions. A non‐targeting siRNA (siGENOME Control Pool Non‐Targeting siRNA, Thermo Fisher Scientific) was used as a negative control. Cells were incubated for 48 h post‐transfection before proceeding to downstream analyses, including proliferation and migration assays. All experiments were performed in triplicate.

### 2.12 Cell Proliferation Assay

The effect of MMP3 and POSTN knockdown on cell proliferation was assessed using the Cell Counting Kit‐8 (CCK‐8) (C0038, Beyotime Biotechnology Co. Ltd., Shanghai, China), following the manufacturer's protocol. Briefly, transfected cells were seeded into 96‐well plates at a density of 5 × 10^3^ cells/well and incubated at 37°C in a humidified incubator with 5% CO₂. After 48 h, 10 μL of CCK‐8 solution was added to each well and incubated for an additional 2 h. The optical density (OD) was measured at 450 nm using a microplate reader (DR‐200Bs, Diatek, China). Cell proliferation was expressed as the percentage of absorbance relative to the control group. All experiments were performed in triplicate.

### 2.13 In Vitro Wound Healing Assay

To assess the effect of MMP3 or POSTN knockdown on cell migration, a wound healing assay was conducted 48 h after siRNA transfection. Transfected cells were seeded in 6‐well plates and cultured until ~90% confluence. A uniform scratch was made across the cell monolayer using a sterile 200 μL pipette tip. The detached cells were removed by washing twice with PBS, and serum‐free medium containing 10 μg/mL mitomycin C (Sigma‐Aldrich, Shanghai, China) was added to inhibit cell proliferation. Images of the wound area were captured at 0 h and 48 h using an inverted phase‐contrast microscope (Olympus Corporation, Tokyo, Japan). The wound area was quantified using ImageJ software, and the migration rate was calculated as the percentage of wound closure compared to the initial scratch area. Each condition was analyzed in triplicate, and representative fields were imaged.

### 2.14 Statistical Analysis

Statistical analyses were conducted using the R Studio program (version 4.2.2). Experimental data were reported as mean ± SD and analyzed with SPSS software version 20. Pairwise comparisons were performed using the Wilcoxon rank‐sum test, and *p* < 0.05 was assumed to be statistically meaningful.

## 3 Results

### 3.1 Identification of 156 Differentially Expressed MRGs (DE‐MRGs)

**FIGURE 1:**
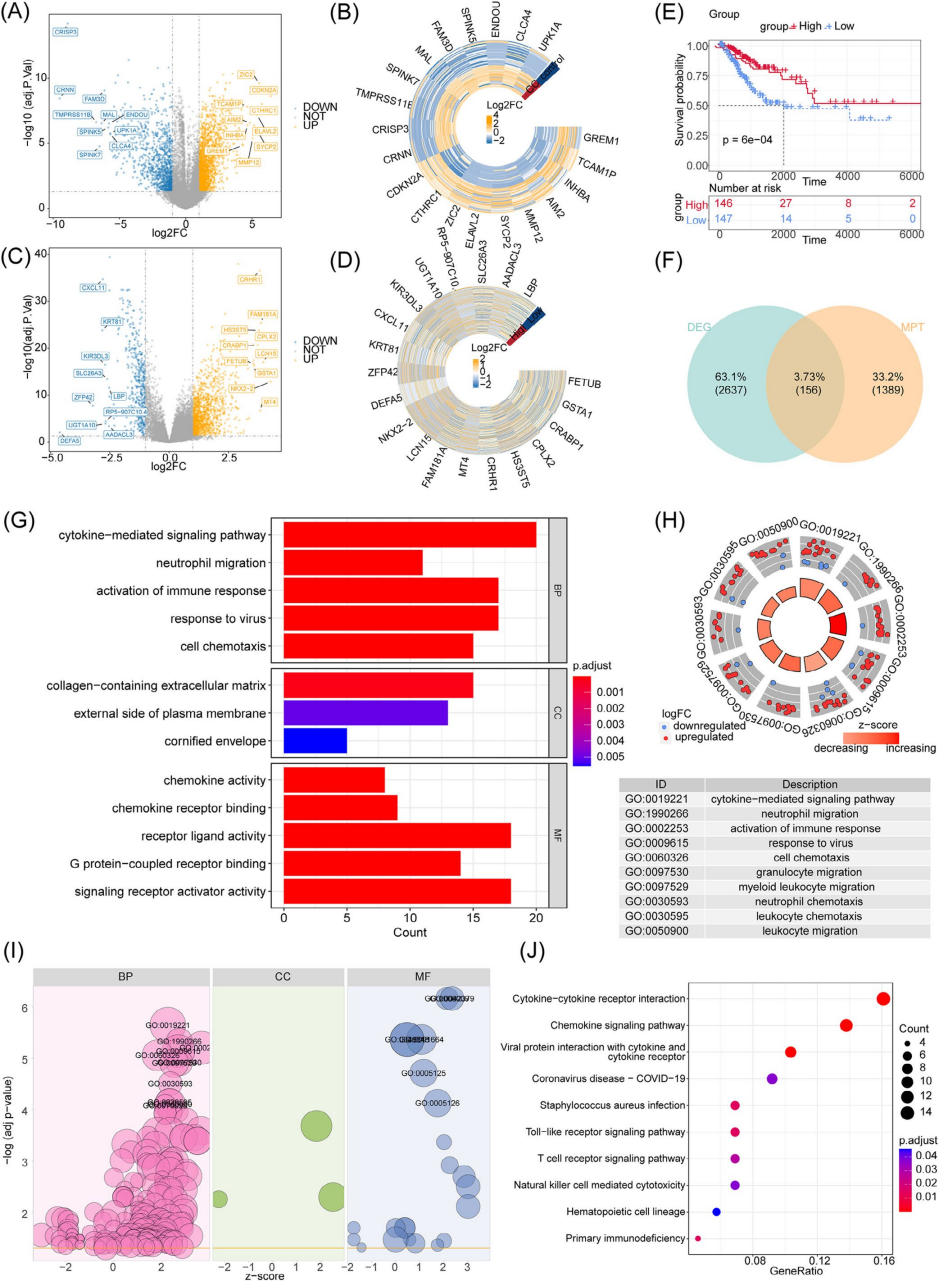
Identification of Mitochondrial permeability transition (MPT)‐driven necrosis‐related differentially expressed genes (DEGs) between the normal and cervical cancer (CC) in GSE63514. (A, B) The volcano plot and heatmap of DEGs1 between the CC and normal groups in the dataset GSE63514. (C, D) The volcano plot and heatmap of DEGs2 between the two scoring groups based on MPT‐driven necrosis‐related gene (MRGs). (E) The CC and normal groups survival analysis. (F) Venn diagram of the intersection of DEGs1 and DEGs2. (G–J) The Gene Ontology (GO) and Kyoto Encyclopedia of Genes and Genome (KEGG) analysis of DE‐MRGs in CC. The bar plot (G), circular plot (H) and bubble plot (I) of the GO pathways enriched for the DE‐MRGs. (J) The result of KEGG pathways enrichment analysis.

A total of 156 DE‐MRGs were identified. Initially, we detected 2793 DEGs between CC and normal groups in the GSE63514 dataset, with 1764 up‐regulated and 1029 downregulated genes (Figure 1A,B). Subsequent analysis of MRGs sorted into high and low‐scoring groups revealed 1545 DEGs, comprising (Figure 1C,D) 1113 up‐regulated and 432 downregulated genes. Survival analysis demonstrated a significant distinction between the high and low‐scoring groups (*p* < 0.05) (Figure 1E). Overlapping DEGs from both analyses resulted in 156 DE‐MRGs (Figure 1F). Moreover, the biological features and signaling channels involved in DE‐MRGs were interrogated.

### 3.2 DE‐MRGs Were Enriched in the Immune‐Related Pathways

The DE‐MRGs were enriched in 277 GO pathways and 143 KEGG pathways, underscoring their involvement in critical biological processes such as immune response activation, cellular chemotaxis, and key immune regulatory signaling pathways, including neutrophil migration and cell chemotaxis (Figure 1G–J).

### 3.3 ICOS, MMP3, and POSTN Were Prognostic Genes

**FIGURE 2:**
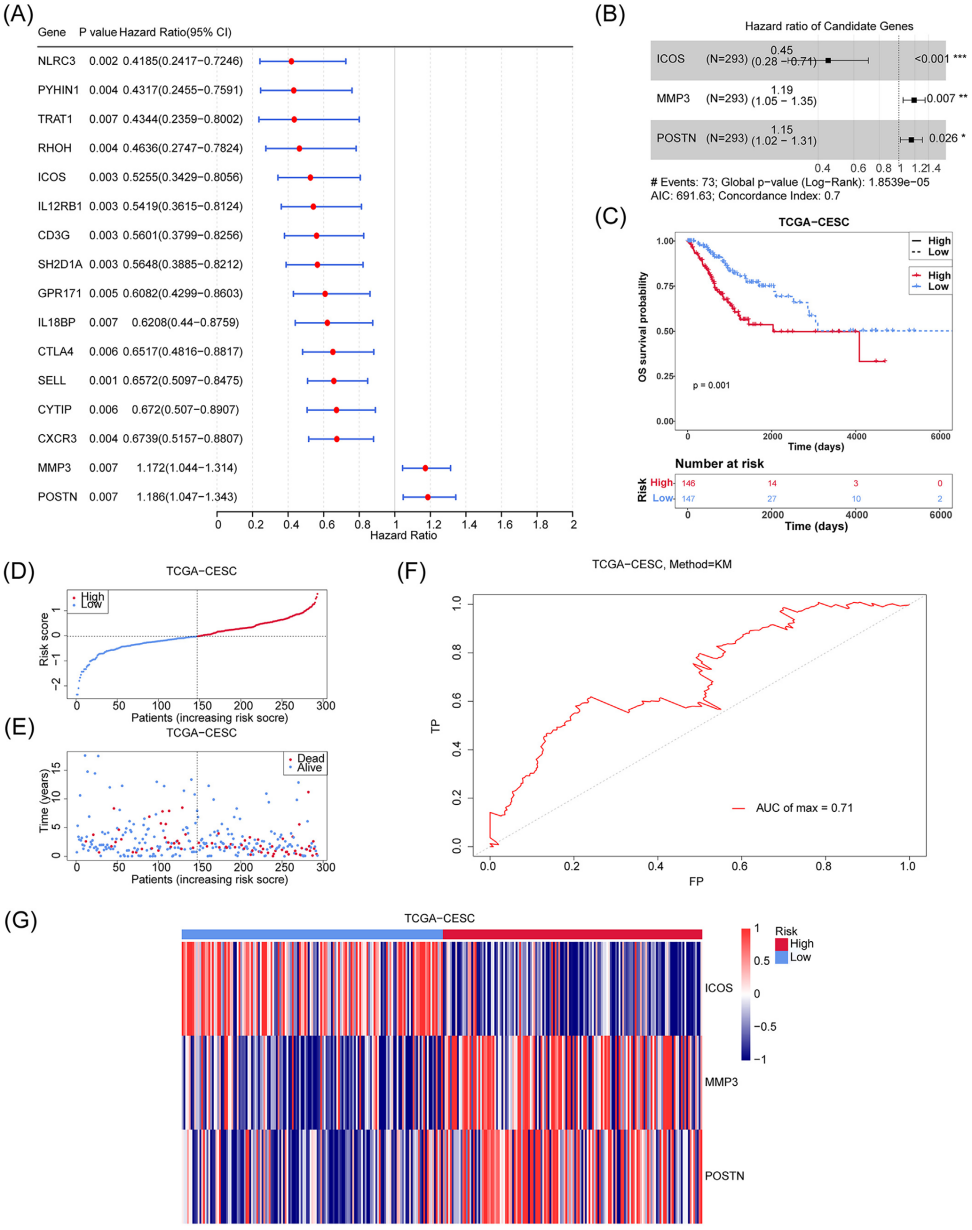
A risk model for outcome prediction in TCGA‐CESC. (A) Univariate cox forest map of prognosis‐related genes. (B) Stepwise regression forest map of prognostic related genes. (C) Kaplan–Meier curves for the overall survival of patients in the high and low risk groups. The high and low risk groups are respectively given in red and blue. (D) The distribution of risk scores for each patient. Red and blue curvles mean the high and low risk, respectively. (E) The distribution of the overall survival status for every patient. Blue and red respectively represent the number of survivors and deaths. (F) The receiver operating characteristic (ROC) curve of the risk score at max time survival. (G) The heatmap of three prognostic genes expressions.

**FIGURE 3:**
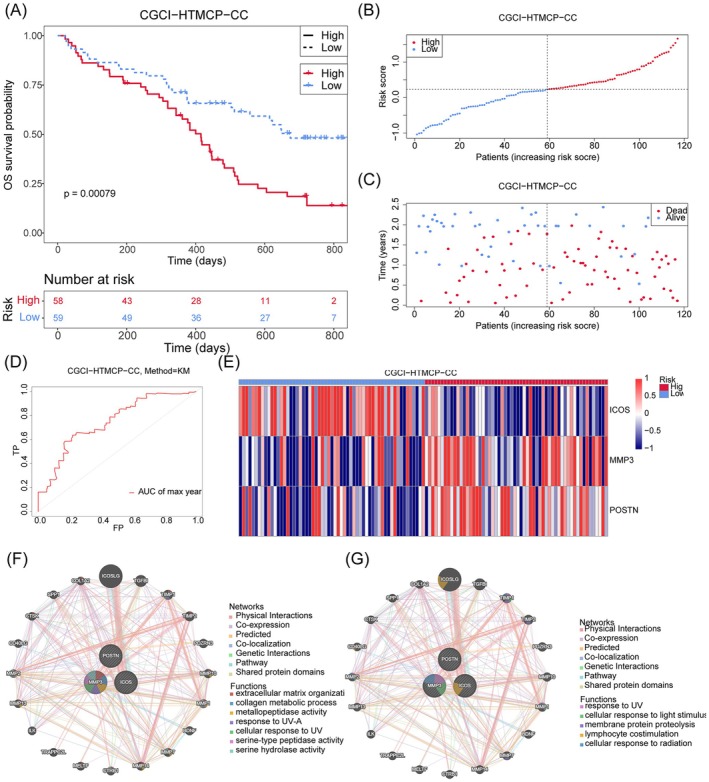
Validation of risk model in CGCI−HTMCP−CC. (A) Kaplan–Meier curves for the overall survival of patients in the high‐ and low‐risk groups. The high‐ and low‐risk groups are respectively given in red and blue. (B) The distribution of the risk scores for each patient. Red and blue respectively mean the high and low risk. (C) The distribution of the overall survival status for every patient. Blue and red respectively represent the number of survivors and deaths. (D) The ROC curve of measuring the predictive value. (E) The heatmap of three prognostic genes expressions. (F, G) Gene–gene interaction (GGI) network of three prognostic genes.

From the DE‐MRGs, 16 genes were identified as related to prognosis. Among these, *MMP3* and *POSTN* are high‐risk genes (HR > 1, *p* < 0.05), whereas *GPR171*, *IL18BP*, *NLRC3*, *RHOH*, *ICOS*, *CTLA4*, *TRAT1*, *CXCR3*, *SH2D1A*, *CD3G*, *CYTIP*, *IL12RB1*, *PYHIN1*, and *SELL* are low‐risk genes (HR < 1, *p* < 0.05). These results suggest that the low‐risk genes may play a protective role in the prognosis of CC (Figure 2A). Following the PH assumption test and stepwise regression, three prognostic genes were retained, *ICOS*, *MMP3*, and *POSTN*, for further analysis (Figure 2B). The risk model, which is a statistical framework used to predict the likelihood of specific outcomes related to tumor progression, recurrence, or treatment response, was then evaluated and validated using the TCGA‐CESC dataset. Our results showed that the survival rate of the high‐risk group was significantly lower than that of the low‐risk group (Figure 2C). The model's reliability was further demonstrated by the finding that higher risk scores correlated with increased mortality (Figure 2D,E), suggesting that the risk model effectively predicts the survival rate of CC (AUC) = 0.71 (Figure 2F). Expression analysis revealed that ICOS was highly expressed in the low‐risk group, whereas MMP3 and POSTN were significantly expressed in the high‐risk group (Figure 2G). These findings were further validated in the CGCI‐HTMCP‐CC dataset, where the high‐risk group showed a significantly lower survival rate compared to the low‐risk group (Figure 3A). Consistent with the previous dataset, higher risk scores correlated with increased mortality (Figure 3B,C), and the risk model showed effective predictive capability (AUC = 0.75) (Figure 3D). ICOS expression remained higher in the low‐risk group, whereas MMP3 and POSTN were more expressed in the high‐risk group (Figure 3E). Additionally, a GGI network analysis using GeneMANIA indicated that the prognostic genes and their interacting partners are primarily involved in lymphocyte co‐stimulation, cellular response to radiation, and serine hydrolase activity, suggesting their potential roles in CC carcinogenesis (Figure 3F,G).

### 3.4 Higher Forecasting Precision of Nomogram Was Probed

**FIGURE 4:**
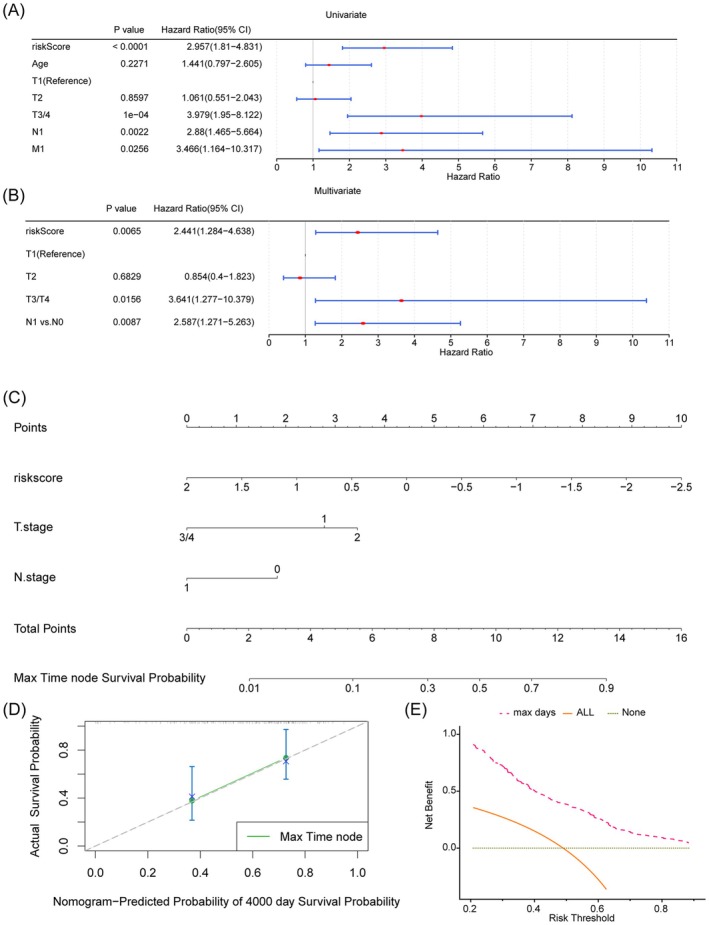
Creation of nomograms based on risk signature combined with clinical characteristics. (A, B) Univariate and multivariate cox regression analysis of the risk signature and different clinical features. (C) A nomogram combining clinicopathological variables and risk score. (D) Calibration curve. (E) Decision curve analysis.

In the TCGA‐CESC dataset, univariate Cox regression analysis revealed that the risk score and T/N/M stages were significantly associated with survival prognosis in CC (Figure 4A). Subsequent PH assumption test and multivariate Cox regression analysis confirmed that the risk score and T/N stage were significantly related to prognosis, leading to the establishment of independent prognostic models (Figure 4B). To provide clinicians with a more precise quantitative method for forecasting the prognosis of CC, a nomogram that integrates risk scores and T/N stages was constructed (Figure 4C). Calibration plots demonstrated that the nomogram was highly predictive of actual survival outcomes (Figure 4D). Additionally, the predictive model, which incorporates various clinical factors, showed a significant net benefit in predicting prognosis (Figure 4E). This indicates that the integrated nomogram is a valuable tool for improving the accuracy of prognosis predictions in CC patients.

### 3.5 TIGIT and LGALS9 Had Remarkable Survival Variances Between the Two Risk Groups

**FIGURE 5:**
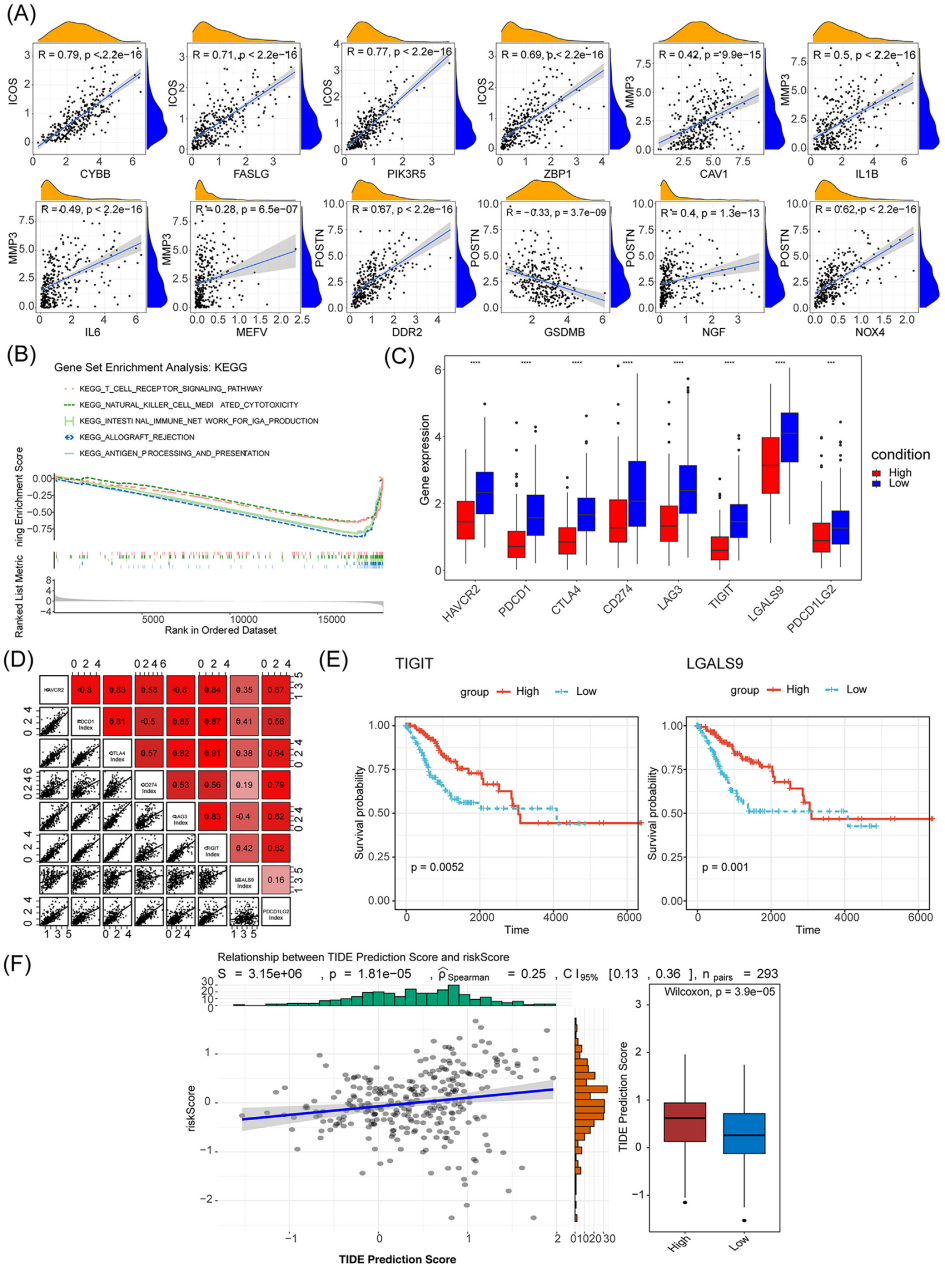
Immune checkpoint differences between the high‐ and low‐risk groups. (A) The correlation between prognostic genes and other categories of cell death. (B) Gene set enrichment analysis (GSEA) results revealed the significantly enriched biological processes between high‐ and low‐risk groups. (C) Difference of gene expression at immune checkpoint between high‐ and low‐risk groups. (D) Correlations between the risk score and different immune checkpoints. (E) The Kaplan–Meier curve survival analysis of immune checkpoints between high‐ and low‐risk groups. (F) Comparison of the tumor immune dysfunction and exclusion (TIDE) prediction scores in the high‐ and low‐risk groups.

Our analysis revealed intricate interactions between prognostic genes and other cell death categories, particularly highlighting positive correlations between ICOS and several genes such as PIK3R5 (*R* = 0.77), ZBP1 (*R* = 0.69), FASLG (*R* = 0.71), and CYBB (*R* = 0.79). In contrast, POSTN was significantly negatively related to GSDMB (*p* < 0.05, R = ‐0.33) (Figure 5A). GSEA offered valuable insights into the signaling pathways involved in CC. The analysis showed that the two risk groups were significantly enriched for 30 signaling pathways including T cell receptor signaling pathways (Figure 5B). Targeting ICP molecules plays a crucial role in regulating immune responses, and their overexpression or dysfunction can lead to immune evasion by cancer cells. Our findings demonstrated that *PDCD1*, a key ICP molecule, and seven other genes, were notably expressed at higher levels in the low‐risk group of cancer patients. This suggests that the upregulation of these genes, including *PDCD1*, *TIGIT*, and *LGALS9*, may be associated with a more favorable immune response and a lower risk of cancer progression or recurrence (Figure 5C). Additionally, significant positive relationships were observed between most of the ICPs (*R* > 0.3) (Figure 5D). Moreover, *TIGIT* and *LGALS9* exhibited remarkable survival outcomes (Figure 5E). A significant distinction in TIDE scores was observed between the two risk groups (*p* < 0.05) (Figure 5F). These results suggest that immune checkpoint blockade therapy might be more beneficial for treating low‐risk CC patients.

### 3.6 Distinct Immune Profiles and Risk Correlations in Tumor Immune Microenvironments

**FIGURE 6:**
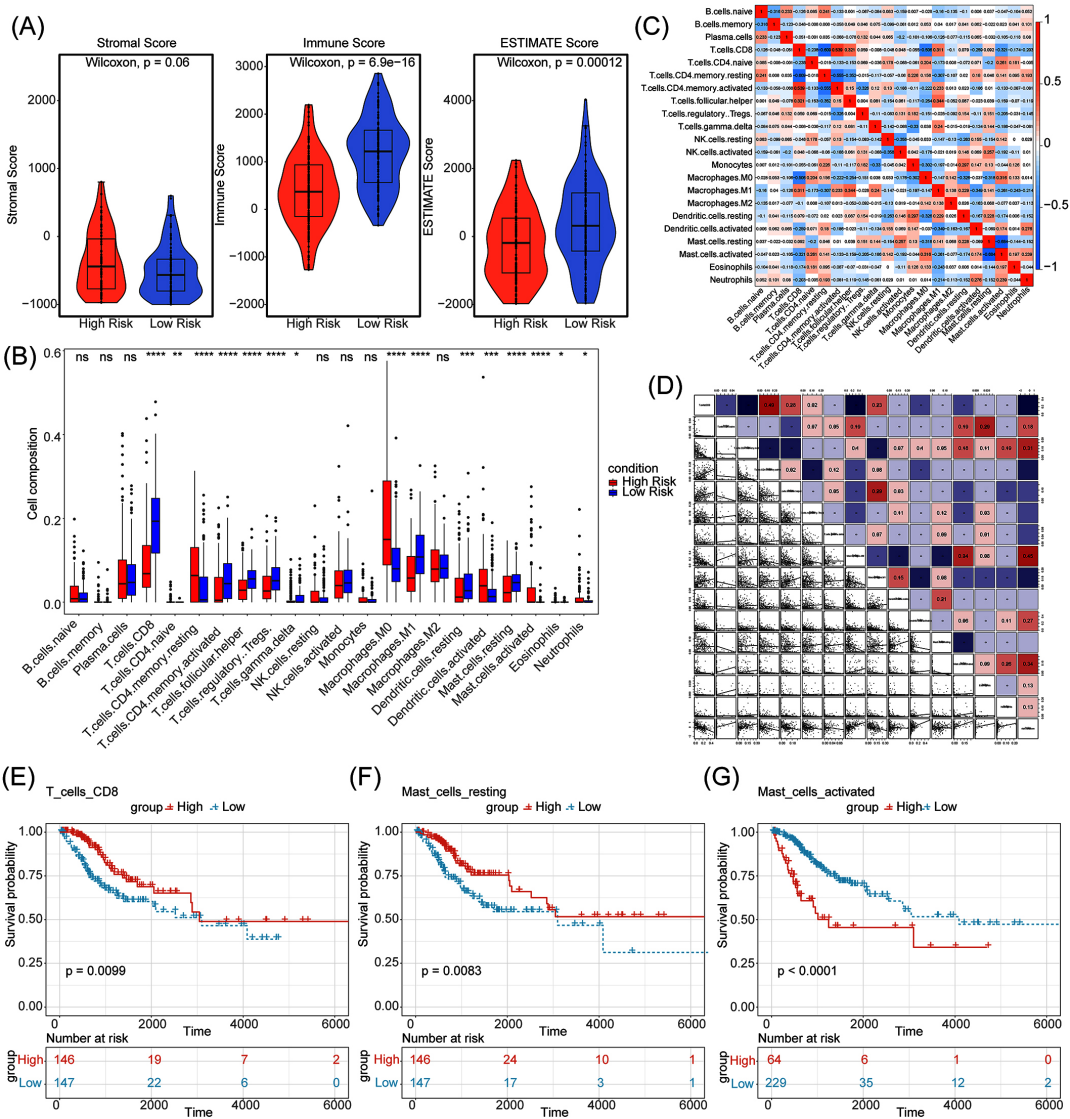
Analysis of the immune cell infiltration landscape in CC patients. (A) Differences among stromal score, immune score, and estimate score between high‐ and low‐risk groups. (B) Difference of 22 kinds of immune cells infiltration between high‐ and low‐risk groups. (C) Correlations among immune cells in CC patients. (D) Correlations between the risk score and differential immune cell profiles. (E–G) The Kaplan–Meier curve survival analysis of three immune cells between high‐ and low‐risk groups. E, F and G represent CD8 T cells, resting mast cells, and activated mast cells, respectively.

Our analysis of the immune microenvironment revealed that the low‐risk group displayed significantly higher immune and ESTIMATE scores compared to the high‐risk group (*p* < 0.05) (Figure 6A), suggesting a more robust immune response in the low‐risk group. We identified significant differences in 15 immune cell types, including CD8 T cells, activated dendritic cells, and activated mast cells between the two risk groups (*p* < 0.05) (Figure 6B). CD8 T cells showed a positive correlation with activated CD4 memory T cells (*p* < 0.05, *R* = 0.54), indicating a coordinated immune response where CD8 T cells eliminate target cells and CD4 memory T cells support and enhance this activity. Additionally, a negative correlation was observed between CD8 T cells and CD4 memory of resting T cells (*p* < 0.05, R = ‐0.61), suggesting a regulatory relationship between these cell types within the immune system (Figure 6C). Macrophages M0 were significantly and positively associated with the risk score (*p* < 0.05, *R* = 0.45), whereas CD8 T cells were significantly and negatively correlated with the risk score (*p* < 0.05, R = ‐0.55) (Figure 6D). To identify immune cells associated with overall survival (OS), Cox regression analysis revealed that CD8 T cells, Macrophages M0, resting mast cells, neutrophils, and activated mast cells were relevant to survival (*p* < 0.05) (Table S2). Kaplan–Meier analysis further indicated significant differences in survival related to CD8 T cells, resting mast cells, and activated mast cells between the two risk groups (Figure 6E–G). These findings underscore the critical role of specific immune cell populations in the prognosis of cervical cancer, highlighting potential targets for therapeutic intervention.

### 3.7 Exploring Differential Drug Responses and Regulatory Networks in Tumor Risk Groups

**FIGURE 7:**
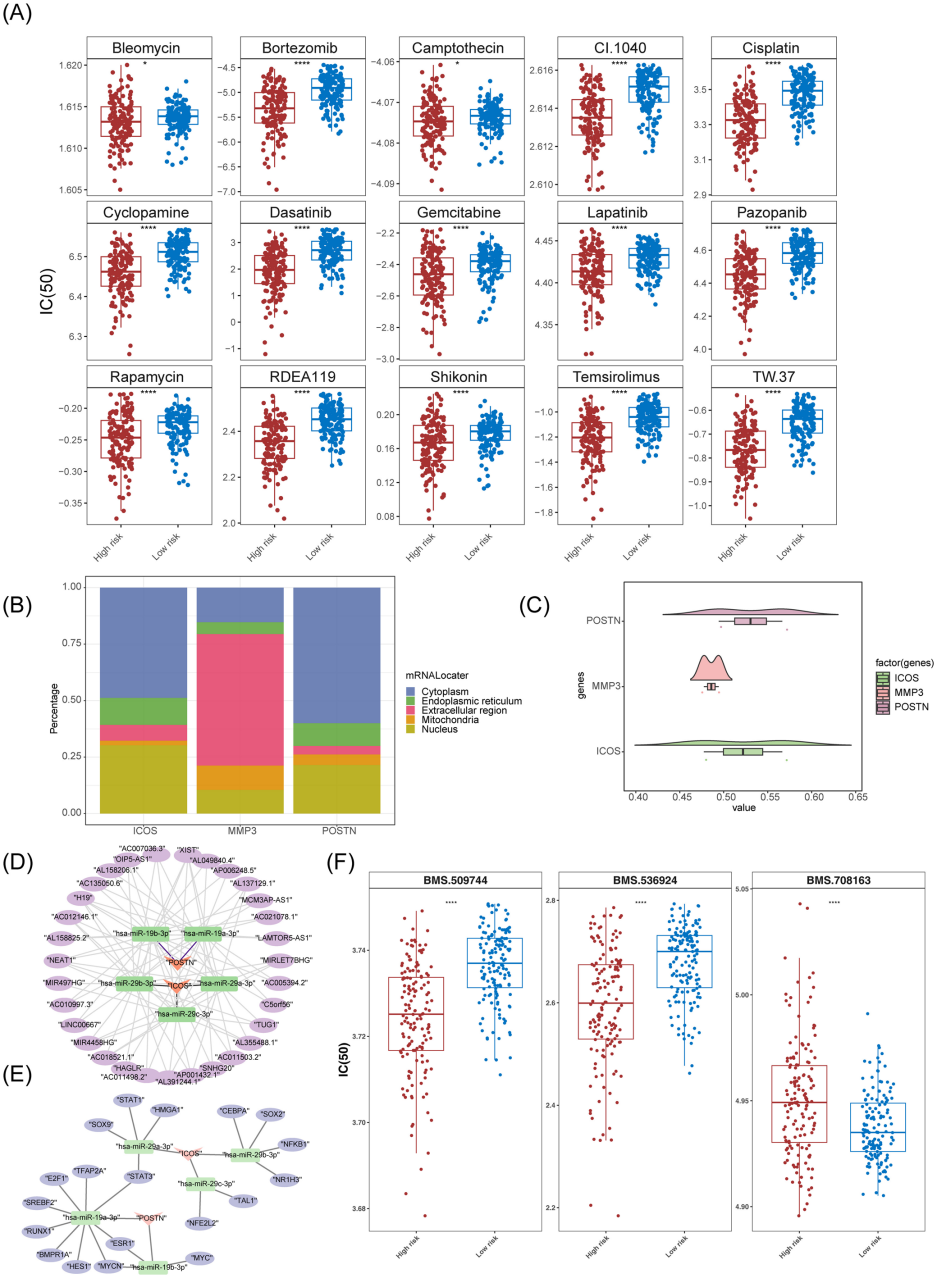
Drug sensitivity and functional analysis of three prognostic genes. (A) IC_50_‐based identification of potential therapeutic compounds for high‐risk patients. (B) Stacked plot depicting the subcellular localization of three prognostic genes. (C) Box plot illustrating the functional similarity of three prognostic genes. (D) ceRNA regulatory network of three prognostic genes. (E) TF‐miRNA‐mRNA network of three prognostic genes. (F) Risk‐Stratified Drug Sensitivity to Immune Checkpoint‐Targeting Agents.

To further explore potential therapeutic responses associated with risk stratification in CC, we estimated the IC_50_ values of anticancer drugs using data from the GDSC database. Notably, several chemotherapeutic agents, including Bleomycin, exhibited significantly lower IC_50_ values in the high‐risk group (Figure 7A, *p* < 0.05), indicating a potentially increased sensitivity to these agents. These findings imply that high‐risk patients may benefit from specific targeted or cytotoxic therapies, which could inform precision medicine strategies. Distinct subcellular localization of proteins dictated diverse biological functions. It was transparent that the subcellular localization of the proteins of ICOS and POSTN was in the cytoplasm, indicating specific intracellular functions for these genes. Conversely, MMP3 was positioned in the extracellular region of the cytoplasm, hinting at a potential role in extracellular matrix remodeling or cell‐to‐cell communication (Figure 7B). Thereafter, the findings in Figure 7C disclosed that ICOS and POSTN shared a high functional similarity. To examine the molecular regulatory mechanisms of prognostic genes, POSTN and ICOS, 5 miRNAs, and 36 lncRNAs were integrated into the network. We uncovered that H19 affected POSTN by regulating hsa‐miR‐19b‐3p (Figure 7D). Additionally, POSTN and ICOS, 5 miRNAs, and 19 TFs were incorporated into the network. We observed that STAT1 modulated ICOS by affecting hsa‐miR‐29a‐3p (Figure 7E). Demonstrates differential therapeutic responses to targeted agents among prognosis‐related genes and immune checkpoint genes, revealing enhanced sensitivity to BMS.536924 and BMS.708163 in the high‐risk subgroup, whereas BMS.509744 exhibited variable efficacy dependent on risk stratification (Figure 7F). In summary, our findings provided insights into the distinct drug sensitivities and regulatory mechanisms of prognostic genes in different risk groups, which may aid in developing more targeted and effective therapeutic strategies.

### 3.8 Determination of Key Cell Types, Genes, and Cell–Cell Communication Pathways

**FIGURE 8:**
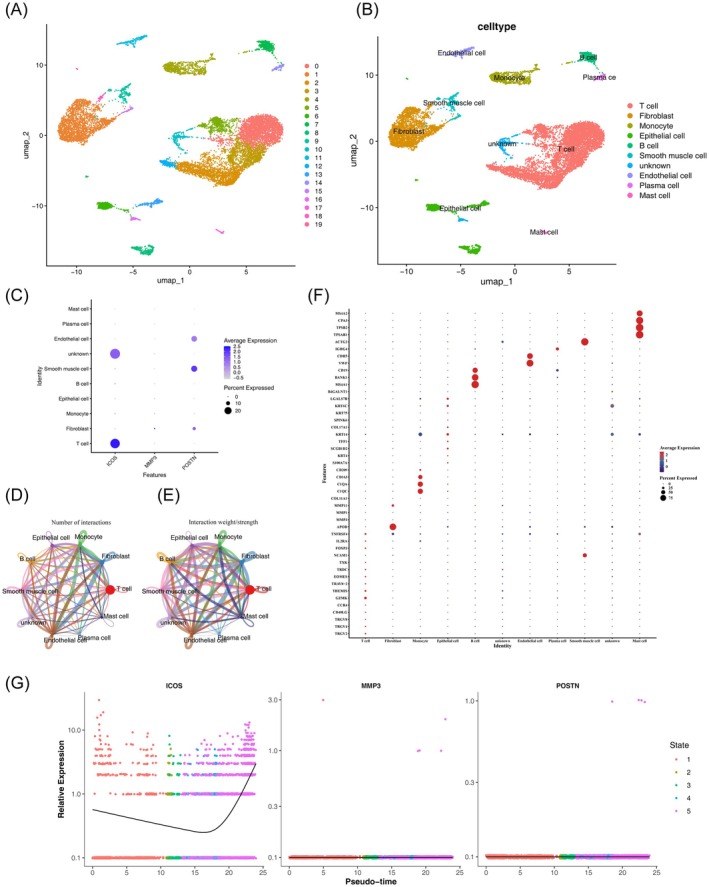
Single‐cell RNA‐sequencing analysis of GSE197641. (A, B) Cluster annotation and cell type identification via Uniform Manifold Approximation and Projection (UMAP). (C) Distribution and expression of three prognostic genes in nine types of cells. Cell–cell communication networks show the number (D) and weight/strength (E) of significant ligand‐receptor pairs in key cell clusters. (F) Pseudotime trajectory depicting five distinct stages of cell developmental states. (G) ICOS expression profiling in T cell subtypes, highlighting elevated levels in NK_T cells.

Single‐cell RNA sequencing (scRNA‐seq) is an unparalleled approach for unveiling tumor heterogeneity and intercellular networks, facilitating a deeper understanding of cancer pathogenesis [22]. In the GSE197641 dataset, 15,805 cells and 17,254 genes were analyzed after quality control (Figure S1A–D). The top 10 genes out of 2000 highly variable genes are shown in Figure S1C. PCA analysis showed no significant outliers (Figure S1E,F). Linear dimensionality reduction identified 20 as the optimal value for cell clustering (Figure S1G). Cell clustering analysis partitioned the highly variable gene scores into 9 distinct cell clusters (Figure 8A). We annotated 9 cell types: T cell, fibroblast cells, monocyte, epithelial cells, B cells, endothelial cells, plasma cells, smooth muscle cells, and mast cells (Figure 8B). After conducting single‐cell clustering, the expression of three prognosis‐related genes in these two samples at the single‐cell level was compared, as depicted in the figure, with ICOS exhibiting the highest expression level under T cell (Figure 8C). We further investigated cell–cell communication interactions, revealing a strong interaction between endothelial cells and monocyte (Figure 8D,E). Finally, the pseudotime analysis results divided the cell developmental states into five distinct periods (Figure 8F). In summary, our comprehensive analysis of the GSE197641 dataset identified key cell types and genes and elucidated crucial cell–cell communication pathways. These findings lay the foundation for further exploration of their roles in disease processes and potential therapeutic targets. Individual clustering analysis revealed that the expression levels of prognosis‐related genes were significantly higher in cervical cancer (CC) cell samples compared to normal cell samples. Furthermore, analysis of T cell subtypes indicated that ICOS exhibited relatively higher expression levels in T cells (Figure 8G).

### 3.9 The Expression Levels of POSTN and MMP3 in CC Were Significantly Higher

**FIGURE 9:**
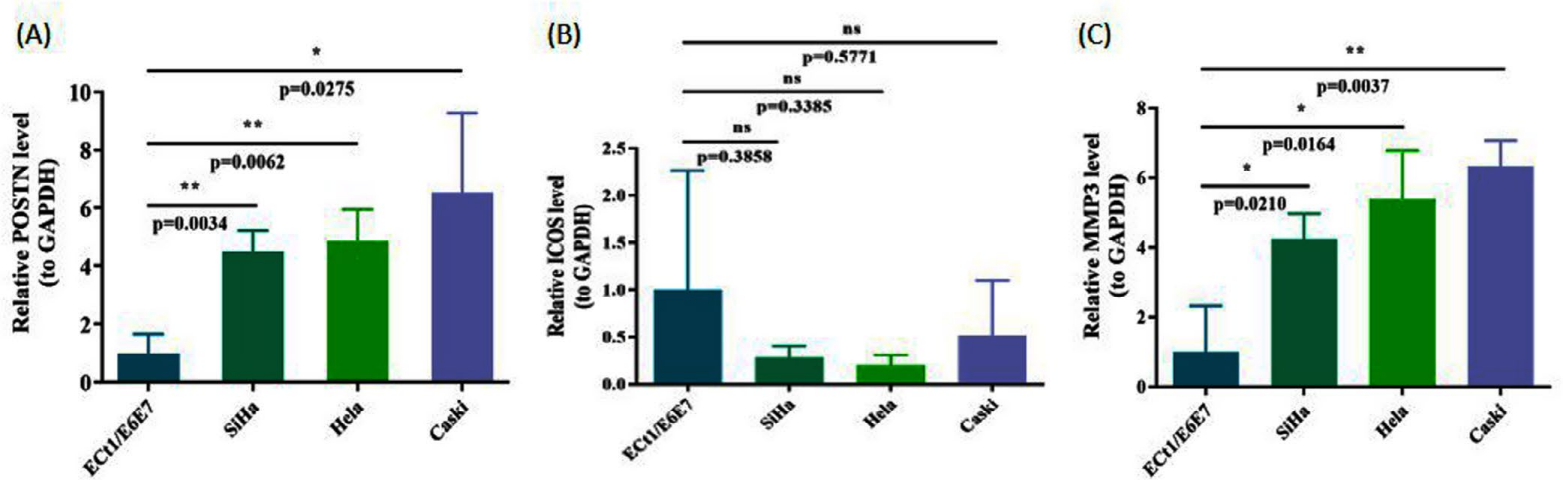
The Reverse transcription quantitative polymerase chain reaction (RT‐qPCR) results of three prognostic genes relative expressing levels in four CC cell lines. (A) POSTN. (B) ICOS. (C) MMP3. The *T*‐tests were performed for statistical analysis.*: *P* < 0.05, **: *P* < 0.01, ***: *P* < 0.001, ****: *P* < 0.0001.

RT‐qPCR validation demonstrated that the expression levels of POSTN and MMP3 in CC tissues were significantly higher than in normal tissues, confirming the reliability of our results (Figure 9, *p* < 0.05). The elevated expression of these genes may greatly impact the pathogenesis of CC.

### 3.10 MMP3 and POSTN Knockdown Inhibits the Proliferation and Migration of CC Cells

**FIGURE 10:**
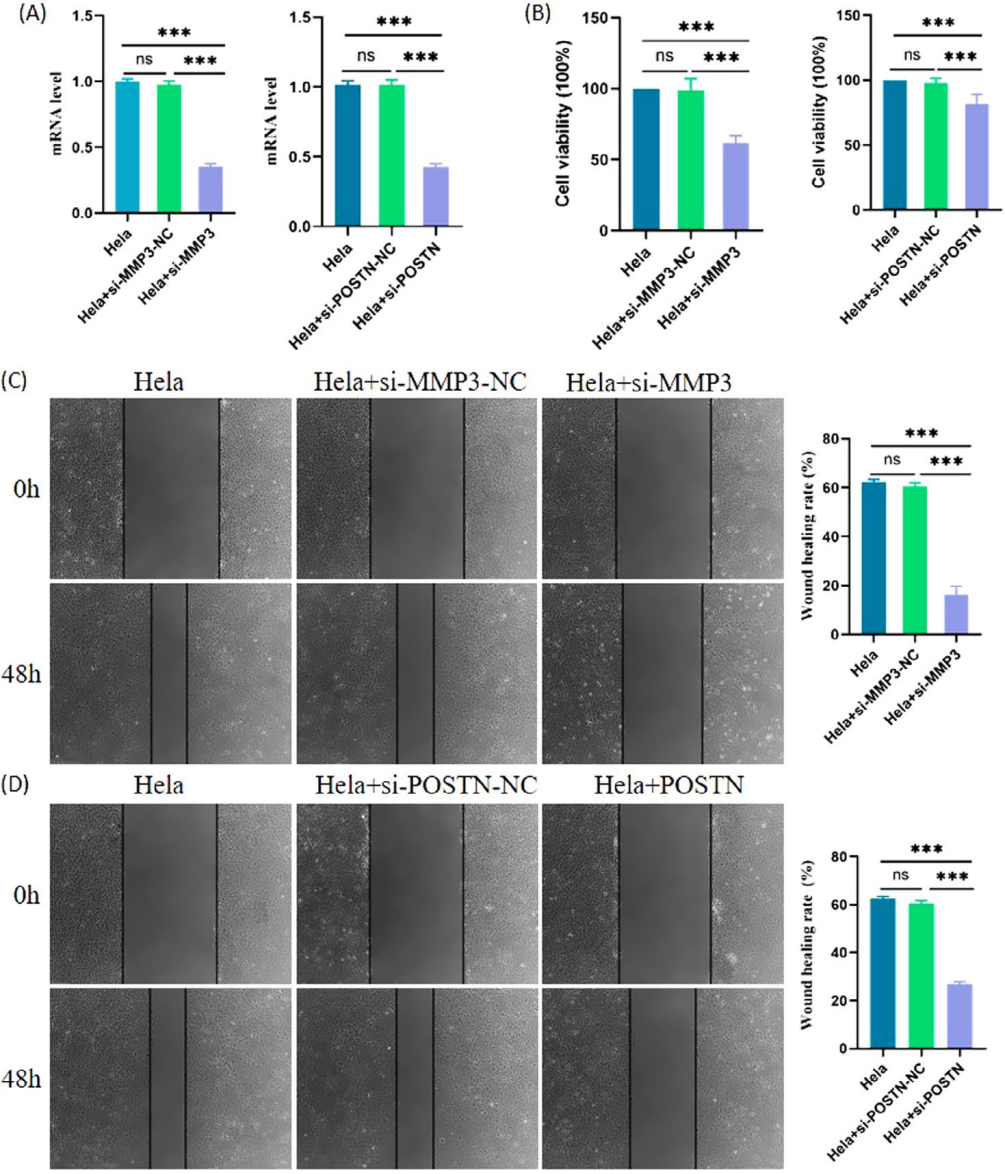
MMP3 and POSTN knockdown inhibited the proliferation and migration of CC cells. (A) After transfection, qRT‐PCR was used to measure the expression of MMP3 and POSTN in Hela cells. (B) The results of the cell proliferation assay showed that the downregulation of MMP3 and POSTN significantly inhibited the proliferation of Hela cells. (C, D) Wound healing assays showed that the downregulation of MMP3 and POSTN significantly slowed the wound healing rate of Hela cells. *: *P* < 0.05, **: *P* < 0.01, ***: *P* < 0.001.

Compared with CaSki cells, HeLa cells are more suitable for proliferation and migration experiments because they are widely used as standard models and are less affected by technical limitations (such as background noise) [43, 44]. To clarify the functional roles of MMP3 and POSTN in CC progression, we performed a series of in vitro assays using HeLa cells. Gene silencing was achieved by transfecting cells with si‐MMP3 and si‐POSTN, along with their respective negative controls. After 48 h, qRT‐PCR analysis confirmed that both MMP3 and POSTN mRNA levels were significantly downregulated in the knockdown groups compared to controls (Figure 10A). Subsequently, functional assays were conducted to assess the impact of MMP3 and POSTN silencing on malignant behaviors. The CCK‐8 assay demonstrated that the knockdown of either gene significantly suppressed HeLa cell proliferation (Figure 10B). In wound healing assays, cell migration was notably reduced in both si‐MMP3 and si‐POSTN groups (Figure 10C,D). Collectively, these results indicate that MMP3 and POSTN play promotive roles in CC cell proliferation and migration. Their knockdown significantly impairs these malignant phenotypes, highlighting their potential as molecular targets for CC therapy.

## 4 Discussion

CC remains the most prevalent malignancy in the female reproductive system, significantly threatening women's health worldwide. Despite continuous advancements in CC treatment, patients with advanced stages of the disease still contend with drug resistance and tumor recurrence, resulting in poor prognosis [44, 45]. Recognizing this, the development of increasingly precise prognostic models has become a top priority for enabling early diagnosis, enhancing predictive accuracy, and ultimately improving treatment outcomes for CC. Mitochondria, the powerhouses of cells and essential regulators of metabolism, play an indispensable role in a wide range of cellular functions [46, 47, 48]. Mitochondria‐driven necrosis, a unique form of programmed cell death caused by mitochondrial dysfunction, has been linked to cancers development and progression. Importantly, this phenomenon has shown immense promise as a prognostic indicator across various cancer types [8, 17, 20]. In the specific context of CC, Sun, Huang, Li, and Luo [45] took a significant step forward by developing a prognostic model centered on six necrosis‐related genes that are integral to the tumor immune microenvironment. This model successfully achieved to precisely predict the overall survival (OS) of CC patients. Building on this foundation, Xing, Tian, and Jin [49] further refined the approach by integrating 16 necroptosis‐related genes and developing a nomogram that integrated risk scores, T stage, and N stage, thereby showcasing robust clinical prognostic capability. Inspired by these advancements, our study has spearheaded the development of a groundbreaking prognostic model that is grounded in mitochondrial‐related genes (MRGs). By exploring the multifaceted role of MRGs in CC, we aim to not only enhance prognosis prediction but also uncover potential therapeutic targets that could substantially enhance the outcomes for CC patients. Bioinformatics methods have significantly advanced the optimization of prognostic models and treatment strategies for cervical cancer. Utilizing tools such as Harmony, Seurat for classical correlation analysis, and LIGER to integrate multi‐omics datasets enables cross‐platform data alignment and enhances model generalizability. For instance, combining TCGA‐CESC (bulk RNA‐seq) with single‐cell data (e.g., GSE197641) can identify tumor‐specific expression patterns, thereby improving the risk stratification of cervical cancer [50, 51]. Based on findings from breast cancer research, where LPS‐stimulated monocytes induce ICAM‐1/VCAM‐1 expression on endothelial cells and anchor cancer cells via β1/β2 integrin‐mediated adhesion, thereby facilitating breast cancer cell metastasis—a mechanistic paradigm that may underpin their crosstalk in cervical cancer [52]. Furthermore, combining immune suppression features derived from scRNA‐seq (e.g., low NCL expression) with somatic mutations (e.g., HPV integration sites) can predict the heterogeneity of immune therapy responses [50].

This study identified three prognostic genes (ICOS, MMP3, POSTN) that may regulate the necrotic process driven by mitochondrial permeability transition (MPT) through multiple pathways. ICOS, as a T cell co‐stimulatory molecule, induces the accumulation of mitochondrial reactive oxygen species (ROS) in tumor cells by promoting the secretion of pro‐inflammatory factors (such as TNF‐α), indirectly activating MPT‐related pathways. Its high expression is associated with enhanced immune responses and improved prognosis in patients with cervical cancer (CC) [53, 54]. MMP3, as a matrix metalloproteinase, mediates extracellular matrix degradation and induces mitochondrial membrane potential collapse through the PI3K/AKT/mTOR signaling pathway [55]; its overexpression can directly cleave mitochondrial membrane proteins (such as CypD) or promote Ca^2+^ overload by inhibiting apoptosis, leading to the disruption of MPTP stability, which is significantly associated with CC invasion and poor prognosis [56, 57]. POSTN inhibits mitochondrial autophagy and exacerbates ROS generation through the integrin αvβ3/FAK/PI3K pathway, triggering irreversible MPT. Its upregulation is closely related to CC metastatic colonization and the formation of an immunosuppressive microenvironment [58, 59]. GeneMANIA network analysis indicates that the three genes and their co‐expressed genes are mainly involved in lymphocyte co‐stimulation, and T cell dysfunction may promote CC progression through immune escape [59, 60]. Although existing evidence supports these genes regulating MPT indirectly through metabolic reprogramming and mitochondrial damage, whether they directly participate in MPTP assembly still needs to be further verified through gene editing and transmission electron microscopy experiments.

Emerging evidence suggests that the interconnectivity of various cell death pathways plays a pivotal role in tumorigenesis [61, 62]. Our findings indicate a positive correlation between ICOS, ZBP1, and FASLG. ZBP1, a DNA‐binding protein, induces cellular inflammatory responses involving pyroptosis, apoptosis, and necroptosis [63, 64]. Recent studies highlight the roles of ICOS and ZBP in RBP‐RNA interactions, affecting the regulation of autoimmunity and auto‐inflammation via different immune pathways [65]. FASLG, a member of the tumor necrosis factor ligand superfamily, is expressed on cytotoxic T cells and induces apoptosis through binding to its receptor, Fas [66]. ICOS and FASLG are favorable prognosis indicators for patients with uterine corpus endometrial carcinoma [67]. Additionally, ICOS ligand and FASLG could suppress tumor growth and extend survival in patients with NSCLC [67, 68]. These findings suggest that the interaction between MRGs and other genes involved in various cell death mechanisms plays a significant role in cancer progression.

It has been documented that MPT‐driven necrosis plays a beneficial role in releasing pro‐inflammatory factors within the tumor microenvironment [69, 70]. We explored the potential mechanism of MPT‐driven necrosis within the tumor microenvironment (TME) and its impact on cervical cancer (CC). Gene Set Enrichment Analysis (GSEA) revealed that the risk signature is predominantly associated with T cell immune pathways, such as antigen processing and presentation, as well as the T cell receptor signaling pathway. Notably, T cells, which are the second most abundant cell type in the TME after tumor‐associated macrophages, play a pivotal role in CC antitumor immunity. Dysregulation or defects in these pathways may allow tumor cells to escape immune surveillance [71, 72]. The findings suggest that enhancing these pathways could improve immunotherapy outcomes for CC. Furthermore, deficiencies in the processing and presentation of antigens triggered by high‐risk human papillomavirus in keratinocytes could exacerbate the postinfection microenvironment, considered a critical factor in the sustained viral presence, dissemination, and malignant progression in CC [73]. Additionally, T cell receptor‐mediated signaling pathways play an essential role in most T cell responses. Dysregulation of TCR signaling can result in anergy or autoimmunity [74]. Moreover, the diminished homodimeric zeta chain molecule, a crucial signaling component in TCR pathways, is associated with stromal invasion in CC lesions [75]. In summary, our findings indicate an intimate association between risk stratification and the T‐cell immune response. The exploration of potential targets within immune pathways holds promise for improving immunotherapy for CC.

Immune checkpoint blockade has recently transformed the management of CC [6]. In our study, we investigated the difference in ICP expression between the two risk subgroups. The findings demonstrated that the most promising therapeutic checkpoints, including PDCD1, CTLA4, and LAG3, exhibited higher expression in the low‐risk group, indicating that patients in the low‐risk group could benefit more from immunotherapy. Moreover, significant positive correlations between most ICPs were observed. Subsequently, TIGIT and LGALS9 were associated with extended overall survival in patients with CC. TIGIT, also known as the T cell immunoglobulin and ITIM domains, emerges as a potential immunotherapeutic target across diverse malignancies. Besides, TIGIT has been identified as a key receptor targeting CD155, a ligand broadly expressed on tumor cells, dendritic cells, and endothelial cells [76]. Binding to CD155, TIGIT suppresses the function of T cells and natural killer (NK) cells, particularly within the tumor microenvironment (TME), where this immunosuppressive axis represents a critical mechanism driving tumor immune evasion. Elevated TIGIT/CD155 expression modulates dendritic cell (DC) tolerance and promotes CD8^+^ T cell exclusion, energy metabolism reprogramming, and exhaustion [77]. Consistent with these mechanistic insights, TIGIT has been linked to poor prognosis in cervical cancer (CC) [78], highlighting its potential role in mediating immune evasion and disease progression in this malignancy. LGALS9 (galectin‐9), a ligand for ICP receptors, has been associated with positive prognosis in colorectal cancer (CC) patients [79]. LGALS9, or galectin‐9, serves as a ligand for ICP receptors, and its elevated expression has been associated with a positive prognosis in individuals with colorectal cancer [79]. Notably, CD44 serves as a receptor for LGALS9 [80], with its expression in tumor and immune cells closely linked to tumor migration, invasion, and immune evasion. The interaction between LGALS9 and CD44 may modulate tumor cell functions, as LGALS9 expression positively correlates with NK cell abundance while negatively correlating with neutrophils. Additionally, this axis demonstrates diagnostic potential for certain diseases, highlighting its dual role in tumor immunoregulation and clinical utility [81]. Furthermore, we explored the correlation between the risk signature and the TIDE score. The high‐risk group had a higher TIDE score and lower immune and estimate scores compared to the low‐risk group, suggesting a greater likelihood of immune escape. These results imply that patients in the high‐risk group may be more prone to developing resistance to immunotherapy.

Tumor immune cell infiltration, a crucial component of the tumor immune microenvironment, plays a pivotal role in the fight against cancer. Our research observed a notable enrichment of activated memory CD8 T cells, CD4 T cells, and M1 macrophages in the low‐risk group, whereas M0 macrophages were enriched in the high‐risk group. Notably, single‐cell RNA sequencing analysis revealed the preferential expression of ICOS, a key prognostic gene, in T cells, designating these cells as pivotal effectors. The upregulation of CD8^+^ T cells enhances adaptive immune responses to cervical cancer vaccines, thereby modulating therapeutic efficacy [82]. M1 macrophages have been reported to exert pro‐inflammatory effects, whereas M0 macrophages promote tumor cell proliferation and invasion [83]. Additionally, CD8^+^ T cells positively correlated with activated CD4^+^ memory T cells and negatively correlated with resting CD4^+^ memory T cells, consistent with their collaborative role in cytotoxic immune initiation [84].

Our study found a positive association between CD8 T cells and improved overall survival (OS) in CC, whereas activated mast cells were linked to poor prognosis. CD8 T cell infiltration, prominent in early stages but decreasing in later stages, is an independent and favorable prognostic factor for CC [85]. In contrast, activated mast cell infiltration correlates positively with angiogenesis, tumor progression, and poor outcomes [86]. These findings confirm the effectiveness of our model and guide personalized immunotherapy in CC.

According to the results predicted by the chemotherapy drug sensitivity analysis, we concluded that CC patients with a high‐risk score might be more sensitive to 15 chemotherapy drugs. Among them, cisplatin was considered the backbone of chemotherapy in CC. Elevated concentrations of cisplatin (800 μM) lead to necrotic cell death, characterized by cytosolic swelling and early loss of plasma membrane integrity in proximal tubular epithelial cells [87]. Dasatinib, a Src inhibitor, can suppress the proliferation of cervical adenocarcinoma cells when combined with anticancer drugs [88]. Diao, Ma, Min, Lin, Kang, Dai, Wang, and Zhao [89] reported that dasatinib promotes paclitaxel‐induced necroptosis in lung adenocarcinoma cells via caspase‐8 dephosphorylation. Bortezomib, a proteasome inhibitor, induces apoptosis in CC cells [90] and enables IFN‐γ‐activated RIP1 kinase‐dependent necrosis in renal cell carcinoma by inhibiting NF‐κB [91]. Additionally, bortezomib triggers both apoptosis and necrosis in human colon cancer DLD‐1 cells [92]. Camptothecin, a broad‐spectrum antitumor drug, exhibits therapeutic effects on various cancers and induces necrosis at higher concentrations in HeLa cells [93]. Cyclopamine, a hedgehog inhibitor, is associated with the abrogation of stem cell properties in CC cells [94]. Blocking hedgehog signaling by cyclopamine can attenuate carcinogenesis in vitro and enhance necrosis in cholangiocellular carcinoma [95]. Additionally, we identified that DNA damage repair‐related drugs such as Bleomycin and Gemcitabine; EGFR inhibitors such as Lapatinib and Pazopanib; PI3K/mTOR inhibitors such as Rapamycin, Shikonin, and temsirolimus; Bcl‐2 inhibitors such as Obatoclax and TW.37; as well as MEK inhibitors such as CI.1040 and RDEA119 with relatively lower IC_50_ values might also be treatment options for high‐risk CC patients. Therefore, it is evident that the model we developed and the prediction of therapeutic drugs had credible evidence.

To gain insight into the underlying mechanisms of prognostic genes, we constructed a TF‐miRNA‐mRNA regulatory network and identified a regulatory axis, STAT1/has‐miR‐29a‐3p/ICOS. ICOS is linked to Th1 responses within T cell‐mediated immune pathways and functions as a sensitizer for chemoradiotherapy in CC [53, 54, 96]. Mutations in ICOS can diminish Th1 cell differentiation by lowering STAT1 activity [97]. This regulatory process may be effectively executed through has‐miR‐29a‐3p. Furthermore, T cells were recognized for their antitumor immune activity in the TME. Notably, cross talk between endothelial cells and monocytes represents another critical axis in tumor progression. Similar findings revealed that LPS‐activated monocytes release TNF‐α to induce endothelial ICAM‐1/VCAM‐1 expression, thereby facilitating breast cancer cell adhesion and metastatic progression. This study constructed a cervical cancer prognosis model based on MPT‐driven necrosis‐related genes (ICOS, MMP3, POSTN) by integrating single‐cell and transcriptomic data. These genes indirectly affect the MPT process by regulating the immune microenvironment (T cell activation), matrix remodeling (ECM degradation), and metabolic reprogramming (ROS accumulation), providing potential targets for personalized treatment. Technically, cross‐platform data integration (Harmony/Seurat) enhanced the model's generalization ability, whereas CellChat analysis unveiled the molecular mechanism by which endothelial cells and monocytes promote metastasis in cervical cancer.

However, there are some problems that should be noted, including sample heterogeneity and insufficient validation: reliance on public datasets (such as TCGA‐CESC) may introduce batch effects, and the lack of independent prospective cohort validation limits clinical translation potential. Mechanism validation is missing: the association between genes and MPT relies on bioinformatics predictions, lacking direct validation through functional experiments (such as gene editing or mitochondrial membrane potential detection). Insufficient control of confounding factors: the impact of HPV subtypes and epigenetic regulation on gene expression has not been fully corrected. Limitations of single‐cell data: a small sample size (such as GSE197641) and lack of coverage of tumor dynamic progression may miss drug‐resistant subgroups. Future studies should combine spatial transcriptomics to analyze the spatially specific expression of genes, validate the potential of targeted therapy using organoid models, and develop dynamic single‐cell technologies (such as Live‐seq) for real‐time monitoring of treatment responses.

## 5 Conclusions

This study comprehensively identifies and analyzes MPT‐driven necrosis in CC for the first time. We identified MRGs with significant prognostic value and developed a novel risk signature that predicts CC prognosis and immunotherapy response effectively. Functional validation further indicated that the silencing of MMP3 and POSTN significantly impedes the proliferation and migration of cervical cancer cells. These findings offer new insights into CC treatment and highlight potential therapeutic strategies. However, limitations exist, such as gene expression validation only at the cellular level. Further studies in animal models and human subjects are needed to fully understand the clinical implications.

## Author Contributions

**Jiaojiao Niu:** methodology, conceptualization, software, writing – original draft. **Sreenivasan Sasidharan:** formal analysis, supervision, writing – review and editing.

## Conflicts of Interest

The authors declare no conflicts of interest.

## Supporting information


**Figure S1:** Quality control and feature selection in single‐cell RNA sequencing analysis. (A) Violin plots displaying the distribution of detected genes (nFeature_RNA), total counts (nCount_RNA), and mitochondrial gene percentage (percent.mt) per cell after quality filtering. (B) Heatmap of the top 10 highly variable genes identified for downstream analysis. (C) Principal component analysis (PCA) plot showing the distribution of cells. (D) Scree plot illustrating the standard deviation of principal components (PCs) used to determine the optimal dimensionality for clustering (PC = 20).

**Table S1:** Primer details for Reverse transcription quantitative polymerase chain reaction (RT‐qPCR).

**Table S2:** Cox regression analysis of immune cells.

## Data Availability

The datasets ANALYZED for this study can be found in the Gene Expression Omnibus (GEO) database (https://www.ncbi.nlm.nih.gov/geo/) and TCGA‐Genomic Data Commons (TCGA‐GDC) (https://portal.gdc.cancer.gov/).
