# Supplementary figures and images for: Development of a Mitochondrial Permeability Transition‐Driven Necrosis‐Related Prognostic Signature in Cervical Cancer: Integrating Bulk Transcriptomic and Single‐Cell Data
Source: Cancer Med. 2025 Aug 1;14(15):e71094. doi: 10.1002/cam4.71094 (PMC12314548; doi:10.1002/cam4.71094)

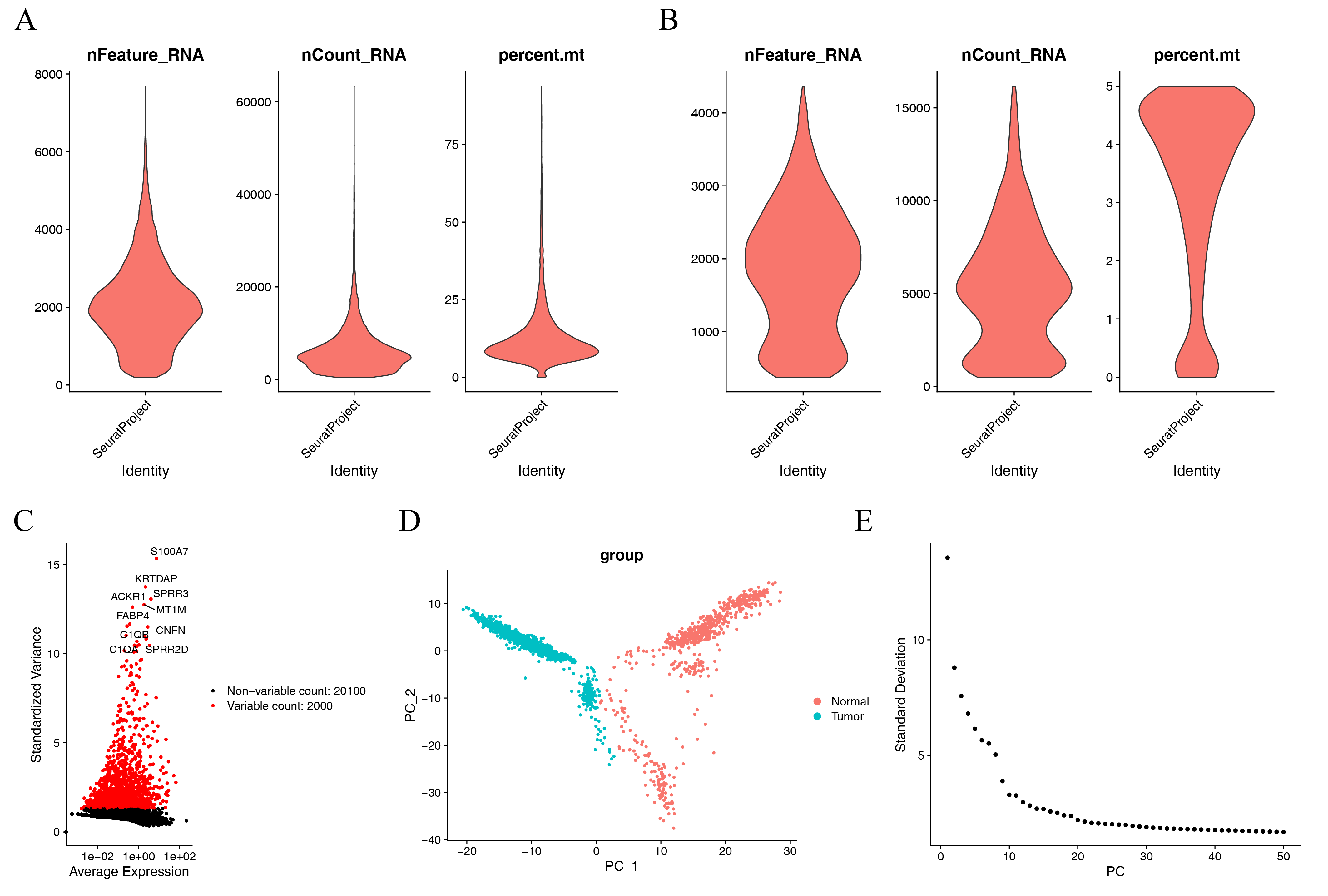

Supplement: Supplementary file 1 — Figure S1: Quality control and feature selection in single‐cell RNA sequencing analysis. (A) Violin plots displaying the distribution of detected genes (nFeature_RNA), total counts (nCount_RNA), and mitochondrial gene percentage (percent.mt) per cell after quality filtering. (B) Heatmap of the top 10 highly variable genes identified for downstream analysis. (C) Principal component analysis (PCA) plot showing the distribution of cells. (D) Scree plot illustrating the standard deviation of principal components (PCs) used to determine the optimal dimensionality for clustering (PC = 20). [file CAM4-14-e71094-s003.png]
