# Supplementary material for: Development of a Mitochondrial Permeability Transition‐Driven Necrosis‐Related Prognostic Signature in Cervical Cancer: Integrating Bulk Transcriptomic and Single‐Cell Data
Source: Cancer Med. 2025 Aug 1;14(15):e71094. doi: 10.1002/cam4.71094 (PMC12314548; doi:10.1002/cam4.71094)
Supplement: Supplementary file 2 — Table S1: Primer details for Reverse transcription quantitative polymerase chain reaction (RT‐qPCR). [file CAM4-14-e71094-s002.doc]

**Supplementary Table S1.** Primer details for Reverse transcription quantitative polymerase chain reaction (RT-qPCR)

| **primer** | **sequence** |
| --- | --- |
| POSTN F | AGAAGACACACCCGTGAGGA |
| POSTN R | CCACAGGAGGCTAACTCCAC |
| MMP3 F | TGAGGACACCAGCATGAACC |
| MMP3 R | ACTTCGGGATGCCAGGAAAG |
| ICOS F | CTCATGTCACTGTGGATGGCA |
| ICOS R | CCAGTCCAAATGCCAGAGCTA |
| internal reference -GAPDH F① | CGAAGGTGGAGTCAACGGATTT |
| internal reference -GAPDH R① | ATGGGTGGAATCATATTGGAAC |
| internal reference -GAPDH F② | CGAAGGTGGAGTCAACGGATTT |
| internal reference -GAPDH R② | ATGGGTGGAATCATATTGGAAC |
