# Supplementary material for: Development of a Mitochondrial Permeability Transition‐Driven Necrosis‐Related Prognostic Signature in Cervical Cancer: Integrating Bulk Transcriptomic and Single‐Cell Data
Source: Cancer Med. 2025 Aug 1;14(15):e71094. doi: 10.1002/cam4.71094 (PMC12314548; doi:10.1002/cam4.71094)
Supplement: Supplementary file 3 — Table S2: Cox regression analysis of immune cells. [file CAM4-14-e71094-s001.docx]

**Supplementary Table S2.** Cox regression analysis of immune cells

| Immune cells | p.value | HR (95% CI for HR) |
| --- | --- | --- |
| T_cells_CD8 | 0.00447 | 0.02833 (0.002429~0.3306) |
| Macrophages_M0 | 0.00919 | 10.69 (1.798~63.55) |
| Mast_cells_resting | 0.00619 | 4.993e-05 (4.157e-08~0.05997) |
| Mast_cells_activated | 5.50E-09 | 66580 (1593~2783000) |
| Neutrophils | 0.0102 | 422600 (21.64~8.255e+09) |
